# Human-Computer Interaction in Smart Environments

**DOI:** 10.3390/s150819487

**Published:** 2015-08-07

**Authors:** Gianluca Paravati, Valentina Gatteschi

**Affiliations:** Politecnico di Torino, Dipartimento di Automatica e Informatica, Corso Duca degli Abruzzi 24, Torino 10129, Italy; E-Mail: valentina.gatteschi@polito.it

**Keywords:** human-computer interaction, smart environments, ubiquitous computing, multimodal systems, natural user interfaces, assistive technologies, mobile computing

## Abstract

Here, we provide an overview of the content of the Special Issue on “Human-computer interaction in smart environments”. The aim of this Special Issue is to highlight technologies and solutions encompassing the use of mass-market sensors in current and emerging applications for interacting with Smart Environments. Selected papers address this topic by analyzing different interaction modalities, including hand/body gestures, face recognition, gaze/eye tracking, biosignal analysis, speech and activity recognition, and related issues.

Sensors continue to rapidly evolve, becoming increasingly smaller, cheaper, more accurate, more reliable, efficient, responsive and also increasing communication capability. These key factors, as well as the availability of new technologies, are contributing to the growth of the market of consumer electronics sensors, thus reducing their costs. This scenario fosters the embedding of sensors in the everyday objects of our lives. The integration of ubiquitous sensing and networking technologies enables the development of new applications in a wide variety of domains such as, among others, smart homes, e-health, and intelligent transport systems.

The aim of this Special Issue is to highlight technologies and solutions encompassing the use of mass-market sensors in current and emerging applications for interacting with the above smart environments.

In particular, the Special Issue gathers together 22 papers concerning current research efforts focused on human-computer interaction (HCI) through natural and intuitive modalities including hand/body gestures [1,2,3,4,5,6], face recognition [7,8], gaze/eye tracking [4,9,10], biosignal analysis [11,12,13,14,15], speech recognition [16], activity recognition [17,18] and their related issues. Moreover, the Special Issue contains papers related to the architectural design of smart environments [19,20], with a particular focus on the exploitation of sensors for gathering information related to the environment [21,22]. In the following, an overview of the papers included in the Special Issue is provided, by summarizing their main contribution to the state-of-the-art.

Smart systems often make use of camera sensors to capture images and video of the user interacting with the system. Digital color cameras can be used as sensing devices for inferring human’s hands position, poses and gestures, to be translated into suitable commands for the control of virtually every kind of digital system. One of the main characteristics of camera-based human-computer interaction systems is the ability to detect and track human gestures and poses in the field of the view of the sensing device.

In this context, a lot of challenges should be faced to guarantee a robust tracking system. In particular, deformable targets and non-rigid objects continuously change their shape and their appearance while being tracked, thus posing additional challenges in the design of such interaction scenarios. Spruyt *et al.* [1] propose a solution to the problem of hand tracking failures by leveraging on context information such as secondary motions of the body or hand-kept objects. More in the details, the proposed methodology suggests analyzing images acquired from a color camera with image processing algorithms to identify and track parts of the body (or other objects) having a given spatial relationship with the hand itself (e.g., the upper arm or something kept in the hand by the user). The position and orientation of the objects avail and give consistence to the results of hand tracking module. 

Adhikarla *et al.* [2] propose a new interaction setup combining glass-free 3D visualization and gesture-tracking technology to provide natural representation and navigation of 3D scenes. Light field displays are used as visualization devices, which provide accurate 3D visualization through directional light beams emitted from 3D scene points. Interaction with 3D scenes is enabled by the analysis of contactless hand and finger gestures. In particular, a “direct touch” methodology for selecting items with a pointing and touching approach is applied. In their paper, the authors describe the proposed haptic interaction framework by focusing on aspects related to the necessary calibration procedure and evaluate the proposed interaction setup by means of objective and subjective tests.

Several kinds of sensors are currently used to give users a natural feeling for controlling computer applications, smart TVs and game consoles. Capturing and recognizing gestures, expressions and vocal inputs led to the evolution of human-computer interfaces giving rise to the development of the so-called natural user interfaces. These recent developments are investigated by Lamberti *et al.* in [3], where a generic framework for a seamless integration of multi-modal natural interaction capabilities is illustrated. The authors address the problem of exploiting the new developed HCI modalities also with applications which are not specifically designed for a natural control. The devised methodology acts as a middleware layer capable of increasing the interaction possibilities for existing applications. Similarly to [3], Lopez *et al.* [4] propose a system for controlling new or already developed applications. In this case, the focus is on mobile devices, where on-board camera sensors provide input for human-computer interaction modules, which enable the control through the analysis of eyes and head movements. 

Kim *et al.* [5] present a system for inferring the pinch-to-zoom gesture in real-time, based on surface EMG (Electromyography) signals. Pinch-to-zoom is a gesture commonly used to control the size of images or webpages on smart devices, and is based on the distance between the thumb and the index fingers. In this work, surface EMG signals are recorded by means of an electrode placed on the first dorsal interosseous muscle and processed by a Support Vector Machine using as feature vectors Welch power spectral density estimates and applying a one-versus-one strategy in order to enable a multiclass classification. As a result, four distance levels (0, 4, 8 and 12 cm) are recognized with a mean correct rate of 93.38%. To verify the usability in a HCI context, the authors exploit the proposed system to remotely control a slideshow and are able to run a 20 min presentation without any errors. Other applications could be smart device control, robot arm control, sign language recognition as well as game applications. 

Camera sensor technology is exploited as an input device for interaction systems also by Lee *et al.* in [7]. The problem faced in this manuscript concerns the implementation of face recognition systems for hardware with low processing capabilities, e.g. Set-Top Box-based Intelligent TVs, where these kind of systems act as the base layer for implementing several kind of services, e.g., automatic log on, family filters (child identification) and provision of personalized advertising. The system takes into account both the limited resolution and quality of the captured images and the low resources provided by Set-Top Box hardware; accordingly, it proposes to decouple the generation of the sensor output from its analysis. Indeed, part of the processing needed to perform face recognition is moved from Set-Top Boxes to *ad hoc* servers which have enough processing power to accurately detect face regions within images.

In the work of Cai *et al.* [8], a new method for improving face recognition systems is proposed. In particular, this work is based on the improvement of Sparse Representation-based Classification algorithms (SRC), which recently proved to be robust and effective in the recognition process. On the other hand, a considerable number of images coming from the camera sensor is needed to obtain good performance. The authors stress the fact that training sets composed by a large amount of samples cannot be practical and propose a method for reducing the training set. Further in the details, the authors propose a solution to the well-known Single Sample Per Person (SSPP) problem in face recognition, where the fundamental requirement for building training sets is that their size is comparable to the features dimension of face samples. Therefore, the authors design a system for modeling intra-class facial variation, which represents an aid for single sample face recognition algorithms to separate frontal/neutral faces from various facial changes. 

Lee *et al.* [9] start from the assumption that the initial delay from user’s commands in web video loading and displaying could negatively affect user experience and propose to exploit gaze and cursor tracking techniques to predict users’ intentions prior to actual command (*i.e.*, click) issued. In this view, they start from the assumption that users tend to move their gaze towards a target before clicking on it, and devise a decision module able to compute the click probability. The proposed system, once it has identified the target area, preloads video data, which will be immediately displayed when users click on a link. 

Eye tracking could also be used as a new information source in intelligent therapies, as suggested by Frutos-Pascual and Garcia-Zapirain [10]. Here, the authors aim at investigating whether a considerable difference in eye-movement patterns could be found among children with different attention skills. In particular, they start from the assumption that participants with better results could have different eye-movement patterns from the ones achieving poorer results. Data related to 32 children interacting with a puzzle game are collected and analyzed. 

Bang *et al.* [11] present a system for assessing eye fatigue caused by 3D displays by analyzing multimodal measurements. Such system collects electroencephalograph (EEG) signals, facial temperature (FT), information on eye blinking rate (BR), as well as subjective evaluations (SE) before and after a 30 min 3D movie watch and evaluates correlation values. In order to measure BR, a remote gaze-tracking system exploiting a high-speed camera has been implemented, whereas facial temperature is acquired by means of a remote thermal camera. Results show that the correlation is high between BR and SE and low between BR and FT. 

New frontiers of HCI are investigated in the work of Tseng *et al.* [12], which introduces a Brain-Computer Interface (BCI) to control multimedia playback based on the user’s physiological state. A multi-channel EEG acquisition module is used to extract a set of features from EEG spectra, which are then analyzed to recognize the user’s physiological state and, accordingly, to propose appropriate music to the listener. Unlike existing systems based on specific activation of mental commands, the interface investigated by the Tseng *et al.* aims to automatically control multimedia playback. The manuscript is focused on the design of the smart multimedia controller and on the analysis of the association between the user’s state and the music selection process.

New types of human-computer interaction involving tactile feedback are investigated by the work of Lee *et al.* [13], which reports on a preliminary study concerning a tactile illusion taking the name “out-of-the-body”. Out of the body tactile illusion refers to a phenomenon which permits the perception of phantom vibro-tactile sensations on the body as if they are emanated from locations which are not directly in contact with *ad hoc* stimulators. In their paper, the authors conduct experiments by analyzing brain imaging and EEG measurements to understand the potential of using this kind of tactile feedback for HCI devices. 

A couple of works included in this Special Issue are related to the exploitation of sensors in smart vehicles, which analyze data concerning driver’s vital parameters. In particular, Zheng *et al.* [14] propose to jointly use palmar perspiration and masseter electromyography sensors to assess truck drivers’ mental stress. In this view, the authors analyze the mental stress of 10 drivers in four situations recreated in a driving simulator: a manual truck driving at around 25 meters gap distance from the preceding vehicle and three automatic truck driving at 12, eight and four meters, respectively (since short distances proved to reduce air resistance and fuel consumption). Results of this study, confirmed by subjective reports, show that mental stress significantly increases when the gap from the preceding vehicle decreases, or during sudden deceleration variations in automatic driving.

In the work by Li and Chung [15], EEG sensors are used to estimate eye closure degree, in order to detect driver drowsiness. In particular, the authors study whether a linear relationship could be found between the eye closure degree and the occipital EEG, being the occipital region the location of the visual cortex, which is responsible for processing visual information. They recruited 20 subjects and let them perform a two-hours monotonous highway driving experiment in a driving simulator. As a result, they found a positive and linear trend of α waves with the growth of eye closure degree and proposed a linear support vector regression model achieving 87.5% and 70.0% accuracy for male and female subjects, respectively. The advantage of the proposed approach, with respect to video-based eye closure recognition systems, is that it is not affected by brightness limitations, and could be used also by drivers wearing glasses in the daytime. 

The interaction with smart spaces assumes a key role especially when sensitive services such as telemedicine and assistive services are considered. Vega-Barbas *et al.* [19] identify the main challenges for the introduction of such services in a Smart Home scenario and, consequently, propose a software architecture modeling the functionalities of the platform needed for maintaining these services. The design methodology is based on activity-theory for developing a user-oriented solution, which addresses a set of acceptability requirements for sensitive services from the point of view of user interaction.

Some works published in this Special Issue deal with the exploitation of sensors for improving human-robot interaction (HRI) [6,16,21]. The underlying idea is that, by relying on information acquired through different sensors, it could be possible to enable a more natural interaction. In this view, Tsuji *et al.* [21] start from the assumption that using only sensors mounted on a robot could not be a suitable choice, since sometimes it could be difficult to gather information on the environment, and present how an intelligent room composed of a network of distributed sensors could be built. To this purpose, room furniture has been equipped with Radio-frequency identification (RFID) readers and load cells in order to accurately identify the position of objects. Movable furniture as well as people movements in the room have been monitored by means of a floor sensing system based on a laser range finder. Data coming from the above sensors has been used by a service robot (responsible for fetching or pointing to objects) which has been equipped with RFID readers and an RGB-D camera, activated when it is close to a region of interest, or under users’ requests. In this work, particular attention is devoted to protect users’ privacy: in this view, the use of vision cameras is limited to specific regions. Similarly, the choice of relying on laser range finder for tracking people movements has been done in order to avoid the exploitation of vision cameras. 

While the above work focuses on the exploitation of sensors for identifying the physical context, Alonso-Martín *et al.* [16] aim at contextualizing the information conveyed during the interaction with a robot, by using information enrichment techniques. Here, the authors describe Maggie, a robotic research platform equipped with a number of visual, auditive and tactile sensors, and present a framework for augmented robot dialog system, enabling the robot to process raw data gathered from the above sensors and to take actions accordingly. In particular, in this paper, the authors focus on optical character recognition as well as on non-grammar automatic speech recognition as a manner to gather users’ inputs. Then, they rely on semantic techniques to extract from users’ inputs information related to their moods, to the main entities and concepts they were mentioning, to the topic they are talking about and to other information such as time or money expressions. The last step of their approach consists in an information enrichment phase, in which additional information concerning detected entities is identified and communicated to the user either in verbal or visual mode. 

Krstinić *et al.* [6] propose to rely on color camera sensors to build interactive interfaces for laser guided robots. These interfaces exploit laser spot tracking methods to extract different shapes from laser traces, which are associated to robot commands. Laser pointers are usually easy to track in controlled and indoor environments because of the brightness of the spot, so they convey enough discriminative features to be identified. Conversely, the authors tackle the issue of designing laser-based interactive systems in outdoor scenarios by using embedded devices with limited computational resources. In this paper, the authors face typical challenges of outdoor scenarios, *i.e.*, brightness and lighting changes, sensor ego-motion as well as computational issues to let the method be used with real-time systems.

Human-computer interaction in aircraft navigation systems plays a key role for enhancing situation awareness and supporting decision-making processes. In this sense, the work proposed by Canino-Rodríguez *et al.* [20] represents a step towards the development of smart air traffic systems where inputs coming from sensors provide the pilot with controller indications as well as surrounding traffic situation through the cockpit. In particular, this work proposes a distributed functional architecture based on a multi-agent approach, with the final aim of sketching a list of guidelines useful in the design of cockpit human-computer interaction. In this sense, this work is consistent with the activity theory for the design of human-computer interfaces.

The work of Parviainen *et al.* [22] shows how mobile phones sensors and radio receiver data could be used to infer the environment in which the user is located, as well as activities (s)he is performing. In particular, the authors propose to use a Bayesian maximum a posteriori classifier based on features extracted from mobile phone’s accelerometer, GPS, WLAN, Bluetooth, GSM/3G cell information and short audio recordings. The proposed method is evaluated on a dataset collected in a real-life trial, and results are compared with the ones provided by other classifiers, such as support vector machines and decision trees. In addition, since a single global model for classifying environment and activities could not be able to deal with individual differences, the authors design an adaptation algorithm based on users’ feedbacks. Such an algorithm proves to be able to improve the classification accuracy.

Finally, this Special Issue contains also two survey works, analyzing the exploitation of sensors in the HCI field. The first work, carried out by Shoaib *et al.* [17], aims at presenting an overview of how mobile phones’ sensors have been used for users’ online activity recognition. Here, the authors analyze 30 studies in which classification is done on mobile phones in real time, and discuss various aspects, ranging from experimental setups, to real-time assistive feedback, to resource consumption analysis, *etc*. by underlying their limitations and proposing recommendations for future research activities.

A second work, by Schneider *et al.* [18], is devoted to present an overview of existing research literature related to the use of sensors in the learning domain. In this paper, the authors analyze and classify 82 works by making reference to the Bloom taxonomy of learning, thus identifying how sensors have been used to support learning in the cognitive, affective and psychomotor domains. Moreover, the authors evaluate how sensors could be used for formative assessment as well as for providing feedback to learners. 
